# Chromosome Microarray Analysis of 3832 Patients over 15 Years Confirms Genome-Wide Copy Number Variation in Patients with Developmental Disabilities Including Autism

**DOI:** 10.3390/cimb48090874

**Published:** 2026-08-28

**Authors:** Santosh Chaval, Sahil S. Tonk, Golder N. Wilson, Vijay S. Tonk

**Affiliations:** 1 Cytogenomic Laboratory, Texas Tech University Health Sciences Center, Lubbock, TX 79430-9406, USA; santosh.chavali@ttuhsc.edu (S.C.); vijay.tonk@ttuhsc.edu (V.S.T.); 2 Department of Pediatrics, Texas Tech University Health Sciences Center, Lubbock, TX 79430-9406, USA; 3 MD & MPH Dual-Degree Program, Texas Tech University Health Sciences Center, Lubbock, TX 79430-9406, USA; sahil.tonk@ttuhsc.edu; 4 Department of Pediatrics, Obstetrics & Gynecology, Texas Tech University Health Sciences Center, Lubbock, TX 79430-9406, USA

**Keywords:** chromosome analysis or karyotyping, chromosome array comparative genomic hybridization, copy number variants, developmental disability, autism, genomic distribution of non-allelic homologous recombination

## Abstract

Ongoing need for chromosome microarray analysis (CMA) characterization prompted the description of all 16,138 copy number variants (CNVs) found in 3832 patients studied from 2009 to 2024, 92% of them with developmental disabilities and/or autism. Detailed reporting shows the overlap of variants qualified as benign (15,083 CNVs, sizes 0.1 Kb–3 Mb) or of uncertain significance (216 CNVs, sizes 11 Kb–20 Mb) with pathogenic CNVs (836, 11 Kb–31 Mb), which are emphasized in most studies. Further distinguishing pathogenic CNVs were 88 recurring microdeletion/duplications and 86 in single patients, with all of the former and 66 of the latter having previous syndrome associations. Diagnoses were provided in 749 (20% of) patients, increasing to 21% among the 2470 patients (2015–2024) with their karyotypes recorded. Diagnoses included 61 known chromosomal syndromes, with CMA confirming or clarifying the abnormal karyotype in 187 (7.6%) or 55 (2.2%). The 90 microdeletions averaged 6439 kb in length (with chromosomes 6, 8, 17, and 22 accounting for most cases), while the 90 microduplications averaged 6895 kb (with chromosomes 8, 14, 17, 22, and X accounting for most cases). Together, these represent an average imbalance of 798,000 nucleotides per patient (0.75% of their genome). Continued reporting that match detailed CNV findings with patient profiles, especially symptom spectra, is needed to optimize CMA potential for presymptomatic diagnosis and therapy.

## 1. Introduction

Sequencing of the human genome [1] has been followed by analysis of its sequence variation across individuals and populations [2,3,4,5,6]. Unexpected was the extent of human DNA sequence variation, particularly the extra (microduplication) or missing (microdeletion) DNA segments [2,3] that are now routinely documented by chromosomal microarray analysis (CMA) [4,5,6,7]. This genome-wide screening for subtle DNA excess/deficiency detects copy number variants (CNVs) measuring from several to thousands of nucleotides (kilobases or Kb), complementing the larger aneuploid segments (>5 million base pairs or 5 Mb) detected by routine karyotyping [8]. These microdeletions or duplications are particularly common in patients with intellectual–developmental disabilities [8,9,10,11,12] and/or autism [12,13,14,15], and their detection improves diagnostic yield from 2–3% by karyotyping [8] to 20–25% by CMA [6,7,8,9,10,11,12,13,14,15].

Although its higher yield led to the endorsement of CMA as a first-line test for patients with cognitive and neurobehavioral differences [16], the wide variation in size and gene content of CNVs generated by non-allelic homologous recombination among repetitive elements necessitates interpretation by circumstance [17,18]. Greater prevalence in affected versus ostensibly unaffected people [19,20,21,22,23,24,25,26], recurrence in patients with similar clinical patterns or syndromes, a size above 1 Mb [1,8,18] and gene content [27] support CNV qualification as disease-disposing (pathogenic) rather having no (benign) or uncertain significance [18,28].

Ongoing difficulties with clinical correlation [29], augmented by the occurrence of multiple CNVs in the same patient [30], justifies continued reporting of CMA results obtained as a function of patient population, array methodology, and time. Here, we describe all CNVs obtained from 3832 patients over 15 years in one laboratory, highlighting their potential for presymptomatic diagnosis [6,7,8,9,10,11,12,13,14,15] and management [7,8] of developmental disabilities. The widespread but selective chromosomal locations [30] of CNVs, along with their synergistic appearance, demonstrate that their benefits for long-term genome dosage/arrangement change compensate for these short-term instances of potential harm [31].

## 2. Materials and Methods

### 2.1. Sample Sources

Array comparative genomic hybridization (chromosomal microarray analysis or CMA) was introduced at the Texas Tech Cytogenomics Laboratory in January 2009, supplementing long-standing cytogenetic testing. This study includes all non-oncology samples processed through June 2024. Most samples were obtained from medical center and private practice physicians across the West Texas region, extending north–south from Amarillo to El Paso and eastward to roughly to Abilene/Midland Odessa. Testing was performed under the supervision of the authors (VJT and SC) within the Department of Pediatrics at Texas Tech University Health Science Center, Lubbock. All analyses adhered to CAP and CLIA guidelines, with compliance verified through biennial inspections.

### 2.2. Sample Analyses

Prior to 2009, the laboratory operated as a standard cytogenetic laboratory, performing chromosome analyses using conventional G-banding methods as previously described [29]. Chromosome microarray analysis was introduced in 2009 as an added test, with karyotyping remaining the preferred initial analysis. Beginning in 2016, automated karyotyping of heparinized peripheral blood or fibroblast cultures was followed by DNA extraction and chromosome microarray analysis using validated protocols [29]. Chromosome microarray analysis platforms evolved as follows: from 2009 to 2015, the EmArray Cyto 6000 44k chip (Agilent Technologies, Santa Clara, CA, USA) with BlueGenome software (Illumina/Novagene, Sacramento, CA, USA); from February 2016 to June 2024, the Cytosure Constitutional 60k CGH array (Oxford Gene Technologies, Begbroke, UK) with Cytosure^®^ Interpret software; and from June 2024 onward, the Agilent GenetiSure Cyto CGH 60k Array with CytoGenomics software (Agilent Technologies, Santa Clara, CA, USA). Arrays were processed according to manufacturer specifications, including hybridization, washing, and scanning on Agilent SureScan systems. CNV detection and interpretation followed ACMG consensus guidelines [18], incorporating probe-level quality metrics, log2 ratio thresholds, assessment for copy-neutral loss of heterozygosity, and correlation with databases of variants in normal [19,20,21] or affected people—Exome aggregate consortium [22], DECIPHER [23], the Database of Genomic Variants [24], and ClinVar [25,26]. Twenty-four CNVs classified as likely pathogenic in the 2016–2024 results were grouped with pathogenic findings for reporting consistency.

### 2.3. Databases

All laboratory samples and reports were entered into a deidentified database under IRB approval (Texas Tech IRB-FY2025-128, exempt status granted 12 March 2025). The database was constructed from pre-existing laboratory systems used for patient–physician reports, billing, and accounting. Data were stored on password-protected computers with access restricted to IRB-authorized laboratory personal and approved student/faculty researchers, in compliance with CAP, CLIA, and HIPPA standards. Identifiable information was limited to laboratory accession number, sex, and age (grouped into 1-year intervals for children under five and five-year intervals for older patients). Karyotypes were entered in ISCN (2020, 2022, or 2024 format, as applicable), and all CNVs were documented by chromosomal band location and base pair coordinates, along with classifications as benign, VUS, or pathogenic, according to ACMG guidelines [18].

### 2.4. Database Analysis and Statistics

Data tallies were generated using Microsoft Excel, employing the search, find, and sort functions to organize CNV counts and classifications (pathogenic, VUS, benign). Descriptive statistics, including means, standard deviations, and frequency distributions, were calculated using Excel’s standard formulas. Statistical significance for group comparisons was assessed using two-tailed *t*-tests for continuous variables (e.g., mean CNV size per patient) and N-1 chi-squared tests for categorical variables (e.g., diagnostic yield differences between cohorts) [32]. A *p*-value of less than 0.05 was considered statistically significant.

## 3. Results

Table 1 summarizes 15 years of CMA data and essentially combines two datasets: results from 2009 to 2015 and from 2016 to 2024, with the totals in data rows 1, 2, and 3. Among the 1524 patients (40% of the total) in the earlier group, 264 (17%) had at least one CNV qualified as pathogenic or diagnostic, while 58 (3.8%) and 1202 (79%) patients had only CNVs qualified as variants of uncertain significance (VUS) and benign (data columns 1–5 in Table 1). In the 2016–2024 group, 485 (21%) of 2308 patients had CNVs qualified as pathogenic, while 89 (3.9%) and 1734 (75%) had only CNVs qualified as variants of uncertain significance and benign. Overall, among the 3832 patients, 749 (20%) had at least one pathogenic or diagnostic CNV, compared with 147 (3.8%) and 2936 (77%) qualified as of uncertain significance and benign. The number of CNVs in these patients included those qualified as pathogenic (accounting for 8.6, 4.2 and 5.2% in the 2009–2015, 2016–2024, and combined patient groups), with benign CNVs accounting for the vast majority (89%, 95%, and 94% in the respective groups).

The higher percentage (8.6%) of pathogenic variants in the 2009–15 group, unaccompanied by more pathologic diagnoses (17%), suggests some selective entry of those variants or fewer defined syndromes when the 2009–15 database was compiled. Note that 123 developmental disability patients (3.7%) had CMA results deemed uncertain (VUS), highlighting the need for continued reporting of CMA series with improved iteration of patient findings.

Diagnoses by combined karyotyping and CMA as compared to those by either alone are shown in Table 2. These results are from years 2015–24 when chromosome results were listed in the laboratory database. Total patients are in the far right column. The 2470 patients with CMA or chromosome results comprise the 2308 for years 2016–24 in Table 1 plus another 162 patients from 2015 (including 119 with chromosome results and 3 with culture failure). Biannual patient numbers are listed in the five columns to the left of total patient column, with the corresponding numbers and percentages (top row) shown for the various categories listed in successive rows (right columns, Table 2). Thus, the patients given a diagnosis by karyotyping or CMA are in the second row. Their total number (b) equals patients with a pathogenic CNV (c) plus those with abnormal karyotypes (e), excluding patients where the pathogenic CNV was confirmed (e.g., a large 21 duplication paralleling karyotype of trisomy 21) (f), patients where the pathogenic CNV clarified the chromosome aberration (e.g., showing microdeletion at the breakpoints of an apparently balanced translocation) (g), and patients with an unrelated CNV in addition to the chromosomal diagnosis (e.g., a pathogenic 17q12 microdeletion in a patient with trisomy 21 karyotype) (h). Patients diagnosed by karyotyping and CMA combined totaled 520 (21% of total patients), with similar percentages for the two-year intervals from 2015–16 to 2023–24 (Table 2, second row).

Note that karyotyping alone gave diagnoses in 267 or 14% of patients overall (far right column, fifth data row), while CMA identified additional diagnoses in 7% of patients, resulting in a 21% overall rate. This number rises to 12% if one includes information added by CMA about chromosome aberrations, such as identifying marker chromosomes (too small for accurate chromosome band identification) or demonstrating the presence or absence of microduplications/deletions in translocations that appear balanced by karyotyping. The ability of karyotyping to show polyploidy, translocations, and levels of mosaicism not demonstratable by CMA [8,18] suggests consideration of a tiered approach to chromosome and CMA testing (see Discussion Section). Note also the increased yield that might come from qualifying more VUS-qualified CNVs (3.1–4.3% of totals in Table 2, last row) as pathogenic using point scores [18].

Looking at the data for the 2-year intervals shows some progression in diagnostic sensitivity, from the 2015–16 (19%) to the 2023–24 (25%) patients (second row, Table 2). Increased percentages of diagnosis are also shown for pathogenic CMA (19 to 25%) and abnormal karyotype results (12 to 19%) across the biannual cases (third and fifth data rows, Table 2). Additional pathogenic CMA findings unrelated to abnormal karyotypes vary from 0 to 6.5% over these years (eighth data row), while the percentage of patients with a benign or VUS CNV accompanying an abnormal karyotype shows a 2.1 to 8.7% increase (ninth data row). Similar percentages of CNVs adding diagnostic information and VUS CMA results across the study period (bottom two rows of Table 2) show that resolving VUS qualifications as benign versus pathogenic has not improved over time.

**Figure 1:**
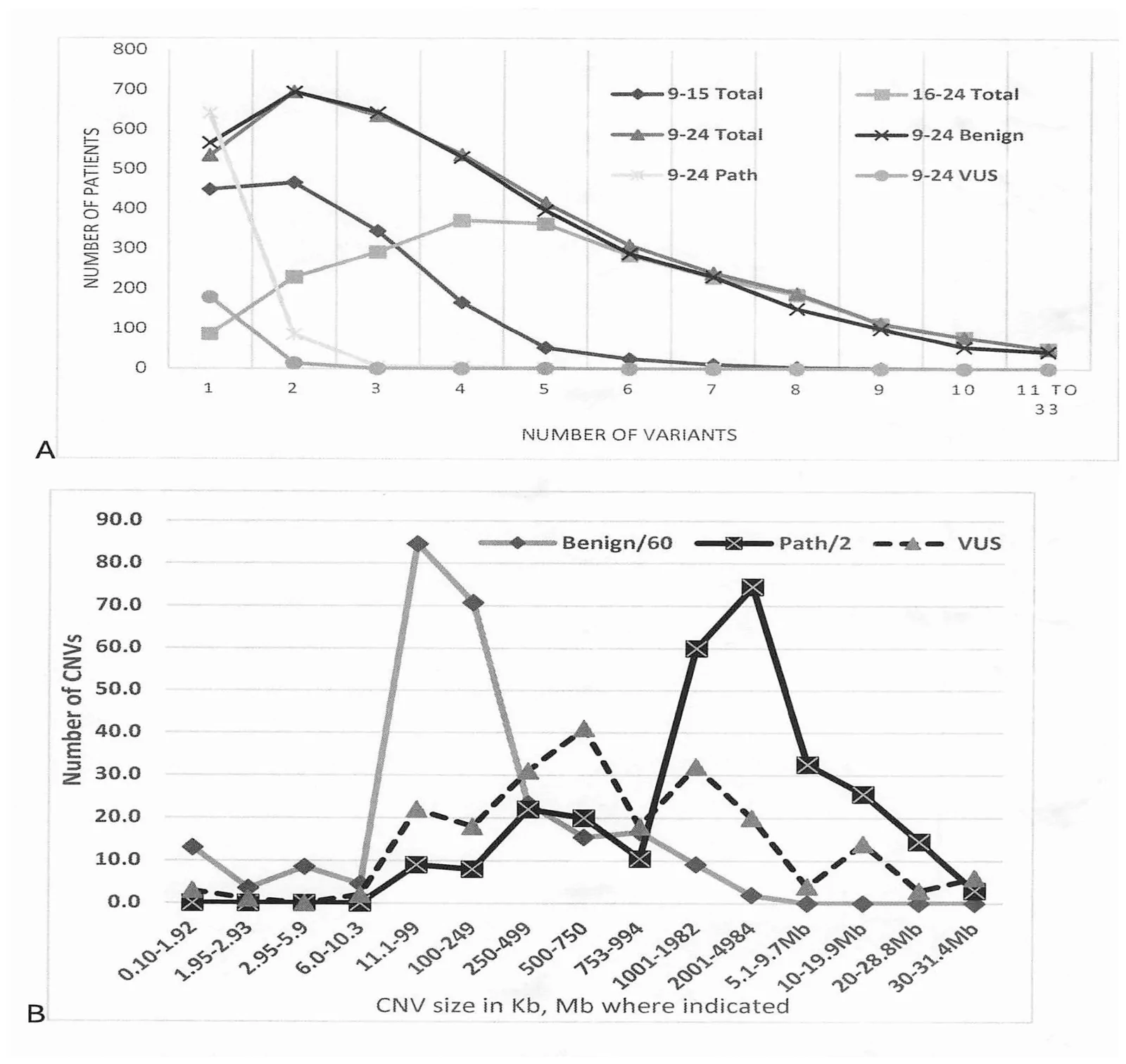
Number per patient and average sizes of CNVs found in 3832 patients from 2009 to 2016. (**A**) Number of variants per patient for total chromosome microarray analysis results 2009 to 2015 (9–15), 2016 to 2024 (16–24), and 2009 to 2024 (9–24). The combined 9–24 results were then tallied by benign, pathogenic, or VUS qualification. (**B**) Distribution of CNV average sizes, with smaller ones in kilobases (Kb) on left, and larger ones in megabases (Mb) on right. Microdeletions and duplications are grouped together in the indicated CNV size intervals.

In Figure 1A, the numbers of variants per patient are shown for the 2009 to 2015 chromosome microarray analyses group (where most had two CNVs), for the 2016 to 2024 group (where most had four CNVs), and the combined group (where most had two CNVs). The greater number of CNVs per patient for the 2016 to 2024 group reflects the change from the older EMArray 44,000 DNA probe chip to the newer Cytosure Constitutional 60,000 DNA probe chip (see Methods Section). Separate plotting of the combined group variants by qualification shows the expected maximum of two per patient for benign variants (94% of all CNVs; Table 1), with numbers of pathogenic or VUS variants declining sharply from the majority of one CNV per patient (Figure 1A, left).

Figure 1B shows the average size of CNVs, with microdeletions and duplications grouped together, ranging from ~100 base pair microduplications and deletions to the ~5 megabase DNA segment size that would be detected by routine karyotyping to the 30 megabase+ aneuploid segments that would be found in patients with traditional trisomy/monosomy syndromes. Note again the majority of benign variants (numbers divided by 60) and the substantial numbers of pathogenic variants (numbers divided by 2). The former peaked at sizes of 11–99 Kb, in contrast to the latter peaking at 2001–4984 Kb or about 2–5 Mb. A challenge in interpreting CMA results is shown by the overlap of CNV sizes for those qualified as VUS (~20 in the 2001–4984 Kb range, ~15 in the 10–19.9 Mb range) and those qualified as pathogenic (right side of Figure 1B). Even a few CNVs qualified as benign had sizes in the 1001–4984 Kb range, reaffirming that size alone cannot be used to qualify CNVs as disease-contributing [8,18].

Diagnoses made by routine karyotyping, sometimes accompanied by fluorescent in situ hybridization (FISH), and/or by CMA, are shown in Table 3. Large CNVs that confirmed aberrations visualized by karyotyping (full or mosaic trisomies, translocations producing large aneuploid segments) are not listed in the right columns. Smaller ones that confirmed, clarified, or added novel CMA to karyotype findings, as enumerated in Table 2, are listed. There were 61 chromosome disorders involving specific chromosomes or bands diagnosed by karyotyping. Another 18 cases, including triploidy, were not listed (see Table 3 legend), but all aneuploid chromosome disorders were confirmed when CMA was performed.

Also correlating with the pathogenic CNVs listed in Table 3 are the lists of specific pathogenic or benign CNVs in Table S1 of the Supplemental Information. Benign variants were selected for display by (1) being in the same chromosome region as a pathogenic variant, and (2) recurring in more than 15 patients as a benign variant. Table S1 thus gives an overview of disease-associated human copy number variation. Exact band loci and average CNV sizes are provided in contrast to Table 3 where overlapping pathogenic variants are grouped. For example, the one 4q32.1/.2 and three 4q32.2q35.2 microduplications in the lower left of Table S1 are grouped as four 4q32.1q35.2 microduplications in the right column of Table 3.

Table 3 thus lists 55 pathogenic CNVs (33 microdeletions, 22 microduplications) found in three or more patients (31 of them in five or more), while Table S1 increases the list to 62 CNVs (37 microdeletions and 25 microduplications) occurring in three or more patients and 36 (24 microdeletions) in five or more. All of the latter have been associated with disability syndromes, as have 66 of 86 (39 microdeletions) occurring in one patient. These numbers foreshadow the large number of disorders with subtle chromosomal imbalances that will be delineated by CMA.

There are equal numbers (90 each) of loci with pathogenic microdeletions or microduplications listed in Table S1. The former occurred in 362 patients and the latter in 222. Average sizes (including one CNV for each locus rather than multiple ones) were 6439 Kb for microdeletion CNVs and 6895 Kb for microduplication CNVs. Loci with 40 or more benign CNVs (shaded in Table S1) included 23 with microdeletions and 26 with microduplications, their average lengths being 263 and 277 Kb, respectively. Size overlap is again shown by microdeletions of 329 Kb (12q23.1) and microduplications of 267 Kb (2p22.1), 338 (4q12), and 387 (4q32.1) qualified as pathogenic versus microdeletions of 1400 kb (5q13.2) and microduplications of 1100 Kb (5q13.2), 1300 Kb (10q11.21), 1100 Kb (18q21.1), and 1300 Kb (Xp22.2p21.3) qualified as benign.

All pathogenic CNVs have loci in common with benign CNVs except for the three at 11q24.1q25 (middle right of Table S1). Slightly more than half (35 of 62) pathogenic CNVs occurred in regions where there were 20 or more benign variants, while less than half (29 CNVs) occurred at loci containing 100 or more benign variants. The 23 loci with multiple benign microdeletions included 15 with pathogenic microdeletions. The 26 loci with multiple benign microduplications included 13 with pathogenic ones. Locations of larger pathogenic CNVs were not significantly associated (*p* < 0.05) with those containing frequent smaller benign CNVs. This indicates that larger, and thus likely pathogenic, CNVs can be generated as primary events rather than as secondary amplifications of benign CNVs.

Adding to the overview of CNVs detectable in the human genome by medical laboratory analysis is the data in Table S2 of the Supplementary Materials, beginning with the distribution by chromosome of benign microdeletions and duplications (columns A–O). CNVs qualified as VUS or pathogenic (216 or 836 variants in Table 1) are not included in these columns because they comprise a small proportion of the 15,083 benign variants; they are included in columns P–Q where the non-overlapping CNV nucleotide lengths and their fraction of the chromosome length are listed. Although CNVs occur on all 24 chromosomes, we see from the conditional formatting in columns C and H that chromosomes 6, 8, 17, and 22 have more microdeletion CNVs (present in 1224, 1016, 601, and 704 of the 3832 patients analyzed, respectively) and that chromosomes 8, 14, 17, 22, and X have more microduplication CNVs (present in 1618, 947, 910, 836, and 1346 of patients analyzed, respectively).

Multiplying these numbers of CNVs (columns C and H) by their average length (columns D, I) gives the number of variant nucleotides in kilobases, which can then be divided by the 3832 patients to give the amount of nucleotide variation per patient (E, J). Dividing nucleotide variation by chromosome length (repeated in columns A, R for reference) gives the nucleotide variation per patient as a proportion of each chromosomal DNA strand (columns F and K). Once average CNV sizes are incorporated, there are more deleted nucleotides on chromosomes 1, 5, 8, and 14 (respective amounts of 33, 60, 21, and 14 Kb) and more duplicated nucleotides on chromosomes 8, 14, 17–15, 22, and X (respective amounts of 94, 98, 40–44, 39, and 50 Kb). Dividing these numbers of variant nucleotides by chromosome length then gives larger percentages of deleted nucleotides for chromosomes 1, 5, 8, 16, and Y (respective percentages of 0.013, 0.033, 0.014, 0.016, and 0.012% in column F) and of duplicated nucleotides for chromosomes 8, 14, 17–15, 22, and Y (respective percentages of 0.064, 0.092, 0.051–0.044, 0.078, 0.032, and 0.042). The bottom row totals in Table S2 indicate that the average patient in this study had 256,000 nucleotides of deleted DNA, amounting to 0.19% of their genome (columns E, F), and 542,000 nucleotides of duplicated DNA, amounting to 0.56% of their genome (columns J, K).

The overall amounts of benign-qualified copy number variations are shown on the right of Table S2. In total, 798,000 nucleotides of copy number change (deletion plus duplication) occupied greater percentages of chromosomes 5, 8, 14–17, 21, X, and Y and constituted 0.72% of the average patient genome (column O). The excess of pathogenic microdeletions (362 versus 222 microduplications) shown in Table S1 is reversed for benign CNVs, which totaled 5984 microdeletions and 9099 microduplications (bottom row of Table S2).

Column N of Table S2 shows that the ratio of total benign microdeletions to microduplications varies widely by chromosome, with numbers 5 and 6 having substantially more microdeletions (darker red conditional formatting), and numbers 1, 2, 10, and 13 having slightly more or nearly as many. The other eighteen chromosomes had more duplications, with numbers 9, 11, 12, and 21 having substantially more (dark blue formatting). When ratios of CNV nucleotides per patient per chromosome length are tallied, chromosomes 1–3, 5, 9, 11, 13, 18, and 20 have more deletions per strand of DNA, while chromosomes 8, 14, 17, 22, and X have an excess of duplications. The contrasting excess of pathogenic microdeletions compared to that of benign duplications fits with the greater impact of deleted DNA known from the phenotypes of chromosome aberrations or autosomal recessive conditions.

A different perspective on CNV genotype/clinical phenotype association is suggested by the data in Table 4, showing that the presence of a (typically larger) pathogenic CNV increases the average number of CNVs in the average patient. Recall from Figure 1B that the different chips used for CMA analysis in the 2016–24 population versus the 2009–15 patients gave more average variants. The numbers of CNVs in patients with the presence or absence of a pathogenic variant are shown for each patient group and all patients. The 3.0, 6.4, and 5.3 mean variants per patient when a pathogenic CNV is present are significantly more than the 2.3, 5.1, and 4.0 when absent at the *p* < 0.001 level. Also increasing average numbers of CNVs per patient is the presence of a large chromosome aberration found by routine karyotyping, raising the oft-discussed possibility that DNA imbalance decreases regulatory constraints on genes within and outside of aneuploid intervals (see Section 4).

## 4. Discussion

Chromosome microarray analysis of 3832 patients identified 16,138 copy number variants (CNVs), the latter reflecting genomic instability driven by multiple repetitive elements, such as low-copy and Alu repeats [2,3,4,5,6,28]. Most CNVs were small and classified as having benign consequences [17,18,28]. Here, benign variants totaled 15,083 (Table 1), with sizes peaking at 11–99 kilobases (Figure 1B). In contrast, pathogenic CNVs—typically larger and mostly in the 2 to 10 Mb range—were associated with developmental disability/autism spectrum disorders [7,8,9,10,11,12,13,14,15] and neurological phenotypes, including epilepsy [11,12], schizophrenia [33], and obsessive–compulsive disorder [34]. These pathogenic CNVs accounted for 836 variants and provided a diagnosis in 749 patients (20% of those analyzed, Table 1).

Among 2470 patients with both karyotyping and CMA results (Table 2 and Table S1 in the Supplementary Materials), 1976 (80%) underwent karyotyping as well as CMA. Abnormal karyotypes were identified in 267 patients (14%), with CMA confirming the karyotype in 187 (70%) and clarifying them in 55 (21%) by revealing subtle imbalances at translocation breakpoints. CMA results added diagnostic information in 304 patients (12%) and identified pathogenic CNVs in 503 (20%), resulting in a combined diagnostic yield of 21%, which is consistent with other published studies [7,8,9,10,11,12,13,14,15].

Since additional karyotype analysis added only 17 diagnoses to the 503 made by CMA when both analyses were used (Table 2), a tiered diagnostic strategy could be considered in certain situations (fetal demise, recurrent pregnancy loss, reimbursement difficulties). This approach would begin with karyotyping, followed by reflex to CMA for the 1772 (90%) with normal karyotypes or ambiguous findings (Table 2 and Table 3), including apparently balanced translocations, complex rearrangements, small deletions/duplications, and marker chromosomes. This combined approach also addresses rare abnormalities, such as mosaicism and triploidy, that typically yield normal CMA results. The latter changes were detected in 24 patients or 1.2% of those with karyotype results.

The high frequency of CNVs documented here and in prior studies highlights their role in genome fluidity and evolution [2,3,4,5,6,30,31]. CNVs provide abundant opportunities for gene rearrangement and separate or clustered gene amplification, as shown by the average 798,000 nucleotides per patient (0.75% of the genome) deleted or duplicated in 3832 patients. These dosage changes were distributed across all chromosomes, with microdeletions more frequent on chromosomes 6, 8, 17, and 22 and microduplications more frequent on chromosomes 8, 14, 17, 22, and X (Tables S1 and S2 in the Supplementary Information). CNV burden per patient ranged from 4–5 variants on average to over 30 in a few (Figure 1A).

Despite this complexity, definitive diagnoses meeting ACMG criteria [18] were achieved for 21% of patients with developmental disability and/or autism, 88 recurring CNVs and 66 of 86 in single patients concordant with published microdeletion/duplication syndromes (Table 3). These CMA diagnoses complemented 61 karyotype-based diagnoses, including Down syndrome (120 cases), trisomy 13/18 (24 cases), and sex chromosome aneuploidies, such as Turner, Triple X, Klinefelter, and XYY syndromes (11, 5, 4, and 5 cases, Table 3).

Diagnostic yield was similar between sexes, with females averaging 4.6 CNVs per patient (19% diagnostic) and males averaging 4.4 CNVs (16% diagnostic; Table 1). Lower yields were found in cases of fetal demise (9.3% diagnostic), familial testing (11%), and infertility (10%). Notable ambiguity concerned Yq11.23 microdeletions in males—two of them were qualified as pathogenic, two as of uncertain significance—and 21 microduplications at Yq11.223/.23 were qualified as benign (Table 3). The latter qualifications contrast with the slightly upstream duplications in the chromosome Y AZFc region at Yq11.221, which are often correlated with azoospermia [35].

The latter ambiguity highlights two major limitations of this study and the general progress of CMA. One concerns the minimal description of patient findings, general terms like developmental/intellectual disability, speech delay, autism, and suspected Down or other syndrome accompanying submitted samples. Detailing the abnormality spectrum from the beginning as a prospective rather than retrospective study would clarify CNV associations since any disability syndrome, from Down to fragile X, can have autistic behaviors [36]. Better phenotypic delineation would likely group many of the 3350 patients with developmental disability (78% of the 3832 total in Table 1, 694 (21%) with pathogenic variants) with the 178 with explicit autism indications (4.6% of 3832, 24 (13%) with pathogenic variants) [8,14,15,36]. A prospective study might also reduce referral bias by setting criteria for CMA testing, as our cohort was undoubtedly biased due to referral of more severely affected patients.

The second limitation concerns a lack of knowledge rather than of information: the relationship between DNA dosage and developmental changes is inferred from circumstantial occurrences rather than mechanisms. These limitations explain why 147 (3.8%) of patients with 216 (1.3%) of all CNVs in Table 1 had qualification results of uncertain significance or VUS, leaving 123 (3.7%) of developmental disability patients with an uncertain diagnostic finding. Aneuploid segment size was not the determining factor, since 70 (32%) of the uncertain results fell within the 1–20 Mb size range of variants deemed pathogenic. Many also occurred in the 11–499 range, where benign variants peaked, or in the 500–1000 bp intermediate region (Figure 1B). Recurrence of CNVs and association with specific abnormality patterns in the literature are major criteria for pathogenesis, showing the value of extending single-center studies like ours to those covering multiple locations.

Using gene content together with size as criteria for pathogenesis is complicated by the variable effects of amplified/deleted genes alone or in contiguity with others [29], especially when multiple CNVs are present. Multiplicity is likely, since the data in Table 4 shows that patients with large pathogenic CNVs or chromosomal aberrations tend to harbor additional CNVs. Data in Figure 1A indicate that 96% of patients in the more recent 2016–24 analyses had multiple CNVs, with 40%, 47%, 10%, and 2% having a respective 2–4, 5–8, 9–10, and 11–33 variants. As with whole-exome sequencing [37], network interactions of genes within CNVs will be important to consider when correlating dosage changes with complex disease.

Ambiguities emphasized by this and other studies argue for more basic research that systematically generates CNVs of different sizes in simpler organisms and cell cultures, correlating effects with size and gene content [38]. Genotype–phenotype registries on the patient side could relate CNV qualities (size, prevalence, affected genes) and their holistically documented clinical findings using large language models of artificial intelligence [39]. As CMA clinical correlations become causal as well as circumstantial, its presymptomatic identification of infants predisposed to disability could revolutionize their treatment [36].

## Figures and Tables

**Table 1:** Chromosome microarray analyses and resulting CNV qualifications by year, sex, and testing indication.

Category (Yrs, Sex, Ind.)	Total Pts (% 09–24)	Total Pts	DNA Dx (% of Pts)	DNA VUS (% of Pts)	DNA nonDx (% of Pts)	Total CNV (% 09–24)	Total CNV (% of Var)	Path (% of Var)	VUS (% of Var)	Benign (% of Var)
2009–2015	1524 (40)	1524 (100)	264 (17)	58 (3.8)	1202 (79) *	3655 (23)	3655 (100)	315 (8.6) *	73 (2.0) *	3267 (89)
2016–2024	2308 (60)	2308(100)	485 (21) *	89 (3.9)	1734 (75)	12,483 (77)	12,483 (100)	521 (4.2)	143 (1.1)	11,816 (95) *
2009–2024	3832 (100)	3832 (100)	749 (20)	147 (3.8)	2936 (77)	16,138 (100)	16,138 (100)	836 (5.2)	216 (1.3)	15,083 (94)
Female	1563 (46) ^1^	1563 (100)	303 (19) **	57 (3.6)	1203 (77)	7117 (47) ^1^	7117 (100)	334 (4.7) **	82 (1.2)	6701 (94)
Male	1845 (54) ^1^	1845 (100)	301 (16)	65 (3.5)	1479 (80)	8140 (53) ^1^	8140 (100)	321 (3.9)	82 (1.0)	7737 (95)
DD-ID-birth defects ^2^	3350 (87)	3350 (100)	694 (21)	123 (3.7)	2533 (76)	13,590 (84)	13,590 (100)	777 (5.7)	178 (1.3)	12,635 (93)
DD-autism	178 (4.6)	178 (100)	24 (13) #	12 (6.7) ^&^	142 (80)	1018 (6.3)	1018 (100)	28 (2.8) #	22 (2.1) ^&^	968 (95)
Fetal demise	161 (4.2)	161 (100)	15 (9.3) #	6 (3.7)	140 (87) ^&^	919 (5.7)	919 (100)	14 (1.5) #	8 (0.87)	897 (98) ^&^
Abnormal relative	123 (3.2)	123 (100)	14 (11) #	4 (3.3)	105 (85) ^&^	556 (3.4)	556 (100)	15 (2.7) #	5 (0.89)	536 (96) ^&^
Other	20 (0.52)	20 (100)	2 (10)	2 (10)	16 (80)	55 (0.34)	55 (100)	2 (3.6)	3 (5.4) ^&^	50 (91)

The 2009–15, 2016–24, and combined (darkly shaded) results of the chromosome microarray analysis are shown in the top 3 rows. Total patients (pts, 1st data column) and CNVs (6th column) are converted to 100% in the lightly shaded 2nd and 7th columns, allowing proportions of diagnoses or variant (var) qualifications to be indicated in columns 3–5 and 8–10. ^1^ Only 3408 patients and their associated 15,257 variants were designated by sex; 424 patients in the 2009–2015 database were entered as sex unknown. ^2^ Many patients had testing indications of developmental (DD) or intellectual (ID) disability and birth defects combined. “Other” includes non-disability diagnoses like Ehlers–Danlos syndrome, male infertility due to azoospermia, and recurrent pregnancy loss. CNV or var, copy number variant; Dx, diagnostic; path, pathogenic; testing, test indication; VUS, variant of insignificance. * Significantly larger (*p* < 0.05) than percentage for years 2009–15 or 2016–24; ** significantly larger than percentage for males; # significantly lower than percentage for patients with DD-ID-birth defects as testing indication; ^&^ significantly larger than percentage for patients with DD-ID-birth defects as testing indication.

**Table 2:** Utility of chromosome microarray analysis in patients with documented karyotype results.

Category	Symbol/Calculation	% Explanation	2015–2016	2017–2018	2019–2020	2021–2022	2023–2024	Total
Patients	a	n/a	483	561	571	533	322	2470
Diagnosis (Dx) by karyotype or CMA	b = c + e − (f + g + h)	(% of pts b/a)	91 (19)	116 (21)	114 (20)	116 (22)	83 (25)	520 (21)
Pathogenic CNV (CMA Dx)	c	(% of pts c/a)	90 (19)	112 (20)	111 (19)	111 (21)	79 (25) *	503 (20)
Karyotype Reported	d	(% of pts d/a)	389 (81)	495 (88) *	434 (76) #	412 (77)	246 (76)	1976 (80)
Abnormal karyotype (Chromosome Dx)	e	(% of karyotype reported e/d)	47 (12)	62 (13)	56 (13)	56 (14)	46 (19) *	267 (14)
Abnormal karyotype (Chromosome Dx)	e	(% of pts e/a)	47 (9.7)	62 (11)	56 (9.8)	56 (11)	46 (14)	267 (11)
Pathogenic CNV confirmed abnormal karyotype	f	(% of pts f/a)	35 (7.3)	41 (7.3)	40 (7.0)	40 (7.5)	31 (9.6)	187 (7.6)
CNV clarifies abnormal karyotype	g	(% of pts g/a)	8 (1.7) #	15 (2.7)	13 (2.3)	11 (2.1)	8 (2.5)	55 (2.2)
Pathogenic CNV unrelated to abnormal karyotype	H	(% of pts h/a)	3 (0.62) *	2 (0.36)	0 #	0 #	3 (0.93) *	8 (0.32)
Benign or VUS CNV unrelated to karyotype	I	(% of pts h/a)	1 (0.21) #	4 (0.71)	3 (0.53)	2 (0.38)	4 (1.2) *	14 (0.57)
CNV adds diagnostic information to karyotype	j = c − (f + i)	(% of cases j/a)	54 (11)	67 (12)	69 (12)	70 (13)	44 (14)	304 (12)
Only VUS CNV found (no diagnosis made)	K	(% of cases k/a)	15 (3.1)	20 (3.6)	21 (3.7)	23 (4.3)	11 (3.4)	90 (3.6)

The far right column shows the total patients. The columns to the left show 2015–24 cases in 2-year intervals; years 2015 and 2024 include data for the last and first six months, respectively. A total of 494 patients had CMA results only (138 with culture failure) and 1976 had chromosome results (data row 4). The second column assigns letters to total cases (a), those with a pathogenic copy number variant (CNV) (c), those with an abnormal karyotype (e), and so forth. The third column provides the percentages. VUS, variant of uncertain significance. * Significantly higher and # significantly lower (*p* < 0.05) biannual case percentage than that of all cases in the far right column.

**Table 3:** Diagnoses by karyotyping and/or chromosome microarray analysis.

Chromosome Dx ^1^	No.	Chromosome Dx	No.	CMA Dx	Del No.	CMA Dx	Dup No.
del(1)(q10)mosaic	2	del(1)(q21.1)mosaic	1	1p36.11/.33	7		
				1q21.1/.2	11	1q21.1/.2	6
dup(2)(q26q27)	1			2p16.1p14	3		
				2q37.1/.3	9		
del(3)(q29qter)	1	dup(3)(q27q29)	1	3q29	6	3p25.2	5
del(4)(p15.2) WHS	2			4p16.3/.1	8	4p16.3/.1	3
						4q32.1q35.2	4
del(5)(p14.1) CduC Sx	2	dup(5)(p15.3)	1	5p15.33p14.3	4		
dup(5)(p15.3)	1			5q35.2/.3	4		
del(6)(q11q16)	1	del(6)(q16.2q21)	1			6q11.1q14.3	3
				7p22.3p21.1	3	7p22.3p21.1	4
del(7)(q11) WS	5	del(7)(q11) WS	2	7q11.22/.23	13	7q11.22/.23	5
				7q33q36.3	3		
Trisomy 8	3			8p23.3/.1	10	8p23.3/.1	6
Trisomy 9	2			9p24.3p23	5		
del(10)(q26.1)	1						
				11q24.1q25	3	11p15.5/.2	3
del(12)(p13.33p11.1)	1						
				13q12.1q13.3	3		
Trisomy 13	11	Translocation 13	1	13q32.3q34	3		
Dup14q24.1q32	1			14q31.3q32.3	4		
Trisomy 15	2	del(15)(q11.2q13) PW/AS	2	15q11.1/.2	27	15q11.1/.2	8
				15q11.1q13.2	16	15q11.1q13.2	5
				15q13.1/.3	9		
				15q26.1/.3	3		
Trisomy 16 mosaic	2					16p13.12/.11	9
				16p11.1/.2	13	16p11.1/.2	9
del(17)(p11.2) SMS	2			17p13.3/.1	7	17p13.3/.1	3
				17p11.2	3		
				17q12	4	17q12	3
Trisomy 18	13	del(18)(q22.3)	1	18p11.32/.21	4	18p11.32/.21	3
				18q21.1q23	6		
						19p13.4/.1	6
Trisomy 21 Down Sx	120	Transloc. Down Sx	10	21q11.2q22.3	6	21q11.2q22.3	5
del(22)(q11.21) DGS	9	dup(22)(q11)	1	22q11.1/.23	41	22q11.1/.23	24
Trisomy 22	2			22q13.2/.3	3		
Turner Sx	11	Turner Sx mosaic	7	Xp22.33p21.3	16	Xp22.33p21.3	9
Triple X Sx	5	Triple X mosaic	3	Xp21.3/.1	4		
Klinefelter Sx	4					Xq26.3q28	9
XYY syndrome	5			Yq11.223/.23	21	Yq11.23	4
Totals	237	61 chromosome Dx	30	33 CMA Dx	282	22 CMA Dx	136

^1^ Not listed are patients with balanced translocations (11), marker chromosomes (4), pericentric inversions (2), or triploidy (10). Only the 55 microdeletions/duplications found in three or more patients are listed; CMA, chromosome microarray analysis; CduC, cri-du-chat; DG, DiGeorge; Dx, diagnosis; PW/A, Prader–Willi/Angelman; SM, Smith–Magenis; Sx or S, syndrome; Transloc., translocation; W, Williams; WH, Wolf–Hirschhorn.

**Table 4:** Increases in CNV numbers in the presence of a pathogenic CNV.

Years	Patients (No.)	CNVs (No.)	Average CNVs/Patient
2009–2015			
All	1524	3655	2.4 ± 1.3
With pathogenic CNV	256	776	3.0 ± 1.6 *
No pathogenic CNV	1268	2879	2.3 ± 1.2
2016–2024			
All	2308	12,483	5.4 ± 2.8
With pathogenic CNV	481	3105	6.4 ± 2.6 *
No pathogenic CNV	1827	9378	5.1 ± 2.8
2009–2024			
All	3832	16,138	4.2 ± 2.2
With pathogenic CNV	737	3881	5.3 ± 2.3 *
No pathogenic CNV	3095	12,257	4.0 ± 2.1
With karyotype aberration	267	1477	5.5 ± 2.6 *

CNV, copy number variant (microduplication or microdeletion). * Significantly different at the 0001 level.

## Data Availability

The raw data supporting the conclusions of this article will be made available by the authors at vijay.tonk@ttuhsc.edu on request.

## Supplementary Materials

The following supporting information can be downloaded at https://www.mdpi.com/article/10.3390/cimb48090874/s1.
