# Supplementary material for: Chromosome Microarray Analysis of 3832 Patients over 15 Years Confirms Genome-Wide Copy Number Variation in Patients with Developmental Disabilities Including Autism
Source: Curr Issues Mol Biol. 2026 Aug 28;48(9):874. doi: 10.3390/cimb48090874 (PMC13605050; doi:10.3390/cimb48090874)
Supplement: Supplementary file 1 [file cimb-48-00874-s001.zip › cimb-4483320-supplementary-Table S1.pdf]

Table S1. Recurring pathogenic and benign CNVs from microarray analysis of 3832 patients

Cband	P-(bp)	P+(bp)	B-(bp)	B+(bp)		Cband
1p36.33/.31	**7(4.5M)**	1(703)	119(412)	84(476)		10p15.3/.1
1p36.2p35.3	0	1(1.1M)	73(473)	108(505)		10p15.3p13
1p33p31.1	1(23M)	0	0	0		10p14p12
1q21.1/.2	**11(1.3M)**	**6(1.9)**	76(617)	51(469)		10q11.21/.22
1q21.1q23.1	0	1(12M)	1(601)	2(1.1)		10q22.3/23.2
1q23.3q25.3	1(18M)	0	2(24)	10(187)		10q26.1/.3
1q31.1q32.1	1(14M)	0	12(308)	13(68)		11p15.5/.2
1q41q44	2(5.0M)	1(27M)	18(180)	26(242)		11p15.1p12
2p25.3/.1	1(2.8M)	1(12M)	2(128)	2(177)		11q13.1q14.1
2p22.1	0	1(267)	0	2(267)		11q22.1
2p16.1p14	**3(232)**	1(2.1M)	10(143)	2(27)		11q24.1q25
2q13q14	1(8.7M)	1(1.2M)	14(203)	33(259)		11q24.3q25
2q14.1q21.1	1(14M)	0	1(526)	2(347)		11q25
2q36.1q37.3	**9(4.8M)**	2(8.1M)	177(133)	31(38)		12q13.1q21.1
3p26.3/p25.2	2(5.5M)	**5(73)**	4(356)	20(188)		12q23.1
3q21.2/.3	0	1(2.8M)	0	7(300)		12q24.1/.2
3q22.2q25.2	2(14M)	0	4(279)	0		13q12.1q13.3
3q26.31q28	1(3.8M)	2(19M)	5(305)	13(80)		13q32.3q34
3q29	**6(1.3M)**	0	5(181)	12(178)		14q11.2q13.1
4p16.3/.1	**8(6.3M)**	**3(3.9)**	61(24)	181(30)		14q31.3q32.3
4p16.3p15.2	0	1(23M)	1(245)	2(519)		15q11.1/.2
4q12	1(338)	0	0	0		15q11.1q13.2
4q21.22/q24	1(22M)	1(578)	1(391)	2(608)		15q13.1/.3
4q32.1/.2	0	1(387)	3(182)	1(255)		15q15.3
4q32.3q35.2	1(15M)	**3(9.8)**	2(722)	13(434)		15q21.3
5p15.33p14.3	**4(13M)**	0	17(210)	22(145)		15q24.1/.2
5p14.1p12	0	2(9.4M)	7(221)	5(524)		15q25.2
5q11.2q13.1	2(16M)	0	1(351)	11(472)		15q26.1/.3
5q13.2	1(855)	0	310(1.4M)	22(1.1M)		16p13.1/.3
5q14.3q21.3	1(8.2M)	1(14M)	3(364)	8(260)		16p12.1/.2
5q33.3q35.1	0	1(21M)	0	5(2.4)		16p11.1/.2
5q35.2/.3	**4(3.0M)**	2(1.2M)	2(355)	3(91)		16q12
6p25.3	1(579)	1(552)	929(54)	255(48)		16q24.1/.2
6p25.3p22.3	1(11M)	1(9.4M)	2(169)	7(494)		17p13.3/.1
6p21.33	0	0	51(36)	47(36)		17p12
6p12.3	1(1.2M)	1(1.2M)	0	0		17p11.2
6q11.1q14.3	0	**3(5.2M)**	1(816)	7(637)		17q11.2
6q16.1	0	1(647)	118(2)	0		17q12
6q21q22.31	1(8.7M)	0	2(312)	0		17q13.1/.3
6q24.1q27	2(5.0M)	2(14M)	3(200)	16(241)		17q21.31
7p22.3/.2	**3(2.3M)**	**3(4.7M)**	1(135)	3(62)		17q24.1/.3
7p22.1p21.1	0	1(17M)	2(197)	9(402)		17q25.3
7q11.21/.23	**13(1.6M)**	**5(1.4M)**	97(95)	280(82)		18p11.32/.21
7q21.3	1(1.6M)	1(1.7M)	5(190)	2(3.6)		18q21.1q23

7q22.3	0	1(1.8M)	*0*	*0*		19p13.4/.1
7q33q36.3	**3(9.4M)**	0	*69(48)*	*82(54)*		19q13.12/.43
8p23.3/.1	**10(6.4M)**	**6(4.8M)**	*911(559)*	*827(342)*		20p12.3/.1
8p23.1p21.1	0	1(15M)	*2(246)*	*7(70)*		20q11.1/.2
8p22p21	1(2.3M)	2(1.2M)	*5(254)*	*36(27)*		20q13.2/.3
8p11.2/.1	0	1(4.3M)	*7(233)*	*26(24)*		21q11.2q22.3
8q12.1	1(557)	1(806)	*1(0.60)*	*535(1.98)*		22q11.1/.23
8q23.1	1(637)	0	*0*	*0*		22q13.2/.31
9p24.3	**4(7.1M)**	0	*4(128)*	*26(127)*		Xp22.33/.31
9p23	1(1.1M)	0	*8(473)*	*11(287)*		Xp22.2p21.3
9p21.3/.1	0	2(551)	*1(345)*	*1(148)*		Xp22.2p11.4
9p13.3/.2	0	1(534)	*0*	*2(156)*		Xp21.3/.1
9q33.2q34.3	1(977)	1(17M)	*4(0.62)*	*49(3.9)*		Xp11.23/.22
Yp11.31/.23	1(27M)	2(27M)	*7(301)*	*11(287)*		Xq11.1q21.1
Yq11.222	13(3.0M)	2(233)	*4(305)*	*2(314)*		Xq13.2
Yq11.223	**3(739)**	1(1.5M)	*3(173)*	*7(225)*		Xq22.3q23
Yq11.223/.23	**15(2.1M)**	**0**	*2(420)*	*30(2.8M)*		Xq24
Yq11.23	**3(877)**	**4(863)**	*3(316)*	*25(302)*		Xq26.3q27.3
Yq12	0	1(270)	*1(18)*	*4(146)*		Xq28

Recurring microdeletion (-) or microduplication (+) CNVs qualified as pathogenic (P) or benign (B-italics) are listed by chromosome band loc

Variants were selected from the total 16,138 based on 1) their qualification as pathogenic or 2) their qualification as benign if 15 or more v

Variants qualified as VUS are not listed, pathogenic variants numbering 3 or more and benign variants numbering 50 or more are highlight

P-(bp)	P+(bp)	B-(bp)	B+(bp)
1(3.0M)	0	*0*	*3(444)*
0	2(15M)	*0*	*5(352)*
2(6.3M)	0	*0*	*1(345)*
0	1(2.4M)	*117(479)*	*29(1.3M)*
2(4.0M)	0	*25(253)*	*15(296)*
2(9.9M)	1(2.3M)	*20(123)*	*105(119)*
1(13.1M)	**3(2.0M)**	*2(352)*	*422(8.8)*
2(17.9M)	0	*0*	*0*
0	1(14.1M)	*9(127)*	*4(17)*
0	1(1.6M)	*0*	*0*
**3(6.3M)**	0	*0*	*0*
0	1(3.2M)	*1(16)*	*0*
2(1.6M)	1(2.1M)	*3(658)*	*9(237)*
1(1.2M)	1(23.2M)	*3(43)*	*8(169)*
1(329)	0	*0*	*0*
1(2.0M)	1(3.7M)	*2(0.51)*	*30(91)*
**3(1.5M)**	1(438)	*17(34)*	*15(35)*
**3(3.8M)**	0	*1(458)*	*6(60)*
1(8.4M)	0	*119(449)*	*18(444)*
**4(4.3M)**	2(3.1M)	*71(274)*	*910(400)*
**27(2.5M)**	**8(2.5M)**	*71(798)*	*123(929)*
**16(3.3M)**	**5(9.3M)**	*73(70)*	*136(310)*
**9(1.7M)**	2(2.1M)	*97(142)*	*116(348)*
1(131)	0	*32(38)*	*29(35)*
0	1(523)	*1(189)*	*2(8.6)*
1(2.6M)	0	*0*	*0*
1(1.4M)	0	*8(410)*	*3(241)*
**3(1.4M)**	2(6.9M)	*4(9.9)*	*13(153)*
**3(642)**	**10(2.3M)**	*15(92)*	*34(39)*
0	1(637)	*49(169)*	*9(324)*
**13(762)**	**9(814)**	*99(975)*	*304(481)*
0	1(479)	*0*	*0*
1(2.6M)	0	*3(124)*	*1(352)*
**7(1.2M)**	**3(4.4M)**	*8(198)*	*10(209)*
2(1.3M)	2(1.3M)	*0*	*2(363)*
**3(3.5M)**	0	*129(146)*	*496(86)*
1(1.0M)	0	*112(13)*	*226(27)*
**4(1.5M)**	**3(1.8M)**	*8(436)*	*117(320)*
1(15M)	1(456)	*0*	*0*
2(503)	0	*342(148)*	*254(117)*
1(4.8M)	0	*0*	*0*
0	1(11M)	*0*	*6(209*
**4(13.1M)**	**3(3.9M)**	*1(40)*	*8(247)*
**6(10.1M)**	2(2.0M)	*6(835)*	*8(1.1M)*

1(4.2M)	**6(13.8M)**	*3(200)*	*8(46)*
0	**3(8.9M)**	*58(245)*	*67(260)*
0	1(1.3M)	*9(161)*	*4(209)*
0	1(1.2M)	*0*	*7(57)*
0	1(11M)	*0*	*14(9.4)*
**6(3.9M)**	**5(11.7M)**	*2(60)*	*49(81)*
**41(2.5M)**	**24(2.0M)**	*700(207)*	*816(180)*
**3(4.8M)**	0	*1(244)*	*11(91)*
**14(2.2M)**	**7(931)**	*280(100)*	*684(97)*
2(25M)	2(1.2M)	*1(212)*	*6(1.3M)*
0	1(24M)	*0*	*11(12)*
**4(2.3M)**	0	*1(133)*	*1(30)*
0	**3(424)**	*38(157)*	*514(160)*
0	2(14M)	*1(101*	*13(116)*
0	1(278)	*0*	*0*
0	2(543)	*0*	*0*
1(10M)	0	*0*	*1(578)*
2(18M)	**3(10M)**	*3(207)*	*8(295)*
1(2.2M)	**6(1.3M)**	*8(90)*	*49(141)*

cation (columns A, G), their numbers and (average length) specified in kilobases or megabases (M).

vere present at that chromosome locus or if a pathogenic variant was at the same locus.

ed in gray.
