# Supplementary material for: Chromosome Microarray Analysis of 3832 Patients over 15 Years Confirms Genome-Wide Copy Number Variation in Patients with Developmental Disabilities Including Autism
Source: Curr Issues Mol Biol. 2026 Aug 28;48(9):874. doi: 10.3390/cimb48090874 (PMC13605050; doi:10.3390/cimb48090874)
Supplement: Supplementary file 1 [file cimb-48-00874-s001.zip › cimb-4483320-supplementary-Table S2.pdf]

Table S2. Number of CNVs and variant base pairs per chromosome from microarray analysis o

	Microdeletion CNVs			
Chrom length (Mb)	Chrom#-del	CNVs reported (no.)	Mean CNV size (X̄ Kb±SD)	Variant Kb per pt
247	1-	239	505±480	33
243	2-	225	189±320	12
200	3-	17	266±180	14
191	4-	94	79±149	3.9
181	5-	344	1,261±506	60
171	6-	1224	57±44	2.5
159	7-	200	95±152	3.9
146	8-	1016	554±333	21
140	9-	21	229±171	8.4
135	10-	169	391±464	14
135	11-	18	219±426	7.7
132	12-	8	99±116	3.4
114	13-	34	249±647	7.4
106	14-	192	383±236	11
100	15-	217	352±613	9.2
89	16-	187	595±701	14
79	17-	601	145±154	3.0
76	18-	15	388±483	7.7
64	19-	61	243±72	4.1
62	20-	10	153±106	2.5
47	21-	2	61±60	0.7
50	22-	704	202±63	2.6
155	X-	363	119±287	4.8
58	Y-	23	467±703	7.1
3080	Total	5984		256

Microdeletion, microduplication, and all CNVs qualified as benign are shown in each section, nur
The aggregate length of all CNVs (variant length) for each chromosome = number of CNVs (colum
The aggregate CNV length (variant length) per patient (columns E, J) = the number of CNVs (colum
Ratios of CNV numbers (columns C/H = column M) and variant kb per patient (columns E/J = N) fo
The variant lengths per patient/chromosome length (columns F + K) are added for deletions and d
CNV coverage in column P represents the aggregate size in megabase pairs of non-overlapping be
CNV coverage over the length of each chromosome is shown in column Q

ɔf 3832 patients

	Microduplication CNVs			
Variant Kb per pt/Chrom length (%)	Chrom#-dup	CNVs reported (no.)	Mean CNV size (X̄ Kb±SD)	Variant Kb/per pt
0.013%	1+	199	404±376	21
0.0049%	2+	101	222±390	5.9
0.0069%	3+	71	233±404	4.3
0.0021%	4+	223	96±177	5.6
0.033%	5+	83	490±547	11
0.0015%	6+	438	75±149	8.6
0.0025%	7+	435	111±232	13
0.014%	8+	1618	223±326	94
0.0060%	9+	115	116±147	3.5
0.010%	10+	173	382±1,016	17
0.0057%	11+	447	20±120	2.3
0.0026%	12+	77	253±1,332	5.1
0.0065%	13+	39	137±217	1.4
0.010%	14+	947	395±316	98
0.0092%	15+	344	495±673	44
0.016%	16+	372	433±494	42
0.0038%	17+	910	168±881	40
0.010%	18+	45	243±474	2.9
0.0063%	19+	101	229±133	6.0
0.0040%	20+	33	127±207	1.1
0.0016%	21+	67	125±168	2.2
0.0053%	22+	836	179±81	39
0.0031%	X+	1346	142±141	50
0.012%	Y+	79	1,184±1,532	24
0.19%	Total	9099		542

ıerical differences highlighted by conditional formatting; Chromosome lengths in millions of base pairs(M

ıns C, H) x mean CNV length (columns D, I).

ıns C, H) x mean CNV length (columns D, I) divided by the number of patients analyzed (3832)

ɔr deletions/duplications are shown for each chromosome

luplications in column O.

ɜnign, pathogenic, or VUS CNVs on each chromosome, CNVs over the Mb resolution of karytype analysis e›

	All CNVs	
Variant Kb per pt/Chrom length (%)	Chromosome #	CNVs reported del/dup
0.0085%	1	1.2
0.0024%	2	2.2
0.0022%	3	0.24
0.0029%	4	0.42
0.0059%	5	4.1
0.0050%	6	2.8
0.0079%	7	0.46
0.064%	8	0.63
0.0025%	9	0.18
0.013%	10	1.0
0.0017%	11	0.040
0.0039%	12	0.10
0.0012%	13	0.87
0.092%	14	0.20
0.044%	15	0.63
0.047%	16	0.50
0.051%	17	0.66
0.0038%	18	0.33
0.0094%	19	0.60
0.0018%	20	0.30
0.0047%	21	0.030
0.078%	22	0.84
0.032%	X	0.27
0.042%	Y	0.29
0.53%	Total	0.66

b) in columns A, Q from https://www.ncbi.nlm.nih.gov/books/NBK22266/, accesse

ҝcluded.

Variant kb per pt/chrom. length del/dup	Total variant Kb per pt/Chrom length (%)	CNV coverage (Mb)
1.6	0.022%	34
2.0	0.0073%	30
3.2	0.0091%	29
0.70	0.0050%	21
5.6	0.039%	34
0.30	0.0065%	40
0.31	0.0104%	27
0.22	0.079%	14
2.4	0.0085%	22
0.80	0.023%	19
3.3	0.0074%	34
0.67	0.0064%	23
5.3	0.0077%	21
0.11	0.102%	17
0.21	0.054%	20
0.33	0.063%	15
0.07	0.054%	36
2.7	0.014%	11
0.67	0.016%	12
2.3	0.0058%	11
0.34	0.0062%	6.6
0.067	0.083%	19
0.10	0.035%	31
0.29	0.054%	7.6
Mean = 1.4	0.72%	535

d 10-23-25;

CNV coverage (% of chrom)	Chrom length (Mb)
14%	247
12%	243
15%	200
11%	191
19%	181
24%	171
17%	159
10%	146
16%	140
14%	135
25%	135
17%	132
18%	114
16%	106
20%	100
17%	89
46%	79
14%	76
19%	64
17%	62
14%	47
37%	50
20%	155
13%	58
17%	3080
